# Incorporating Uncertainty Into the Ranking of SPARROW Model Nutrient Yields From Mississippi/Atchafalaya River Basin Watersheds^1^

**DOI:** 10.1111/j.1752-1688.2009.00310.x

**Published:** 2009-04

**Authors:** Dale M Robertson, Gregory E Schwarz, David A Saad, Richard B Alexander

**Keywords:** statistical methods, ranking, Mississippi, load, yield, nutrients, Gulf of Mexico, hypoxia

## Abstract

Excessive loads of nutrients transported by tributary rivers have been linked to hypoxia in the Gulf of Mexico. Management efforts to reduce the hypoxic zone in the Gulf of Mexico and improve the water quality of rivers and streams could benefit from targeting nutrient reductions toward watersheds with the highest nutrient yields delivered to sensitive downstream waters. One challenge is that most conventional watershed modeling approaches (e.g., mechanistic models) used in these management decisions do not consider uncertainties in the predictions of nutrient yields and their downstream delivery. The increasing use of parameter estimation procedures to statistically estimate model coefficients, however, allows uncertainties in these predictions to be reliably estimated. Here, we use a robust bootstrapping procedure applied to the results of a previous application of the hybrid statistical/mechanistic watershed model SPARROW (Spatially Referenced Regression On Watershed attributes) to develop a statistically reliable method for identifying “high priority” areas for management, based on a probabilistic ranking of delivered nutrient yields from watersheds throughout a basin. The method is designed to be used by managers to prioritize watersheds where additional stream monitoring and evaluations of nutrient-reduction strategies could be undertaken. Our ranking procedure incorporates information on the confidence intervals of model predictions and the corresponding watershed rankings of the delivered nutrient yields. From this quantified uncertainty, we estimate the probability that individual watersheds are among a collection of watersheds that have the highest delivered nutrient yields. We illustrate the application of the procedure to 818 eight-digit Hydrologic Unit Code watersheds in the Mississippi/Atchafalaya River basin by identifying 150 watersheds having the highest delivered nutrient yields to the Gulf of Mexico. Highest delivered yields were from watersheds in the Central Mississippi, Ohio, and Lower Mississippi River basins. With 90% confidence, only a few watersheds can be reliably placed into the highest 150 category; however, many more watersheds can be removed from consideration as not belonging to the highest 150 category. Results from this ranking procedure provide robust information on watershed nutrient yields that can benefit management efforts to reduce nutrient loadings to downstream coastal waters, such as the Gulf of Mexico, or to local receiving streams and reservoirs.

## Introduction

Over-enrichment of nutrients is a major problem in many streams and rivers and can result in the overabundance of benthic algae, phytoplankton, and macrophytes. In addition to local effects, excessive transport of nutrients has been linked to eutrophication of downstream lakes, bays, and estuaries, and also linked to hypoxia in the Gulf of Mexico (USEPA, 2000). Under recommendations of the Clean Water Action Plan in 1998, the United States Environmental Protection Agency (USEPA) developed a national strategy for establishing water body-specific nutrient criteria for rivers and streams, wetlands, lakes and reservoirs, and estuaries (USEPA, 1998). This strategy encouraged states and tribes to establish nutrient standards sufficient to reduce nutrient concentrations and improve the beneficial ecological uses of surface waters. In addition, the Mississippi River/Gulf of Mexico Watershed Nutrient Task Force (2008) established a goal to reduce the size of the hypoxic zone in the Gulf of Mexico to 5,000 km^2^, which will require substantial reductions in nutrient loadings from the Misssissippi/Atchafalaya River basin (MARB).

**FIGURE 1:**
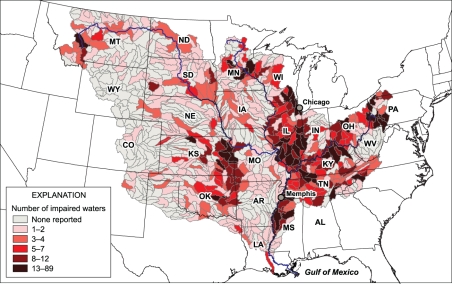
Number of Locations Within Each Eight-Digit Hydrologic Unit Code (HUC8) Watershed Identified as Being Impaired by Nutrient Enrichment (the actual parent cause of impairment was either nutrients or oxygen depletion). Data were obtained from State 303(d) lists submitted to and approved by the USEPA from 2002 to 2006.

To achieve the nutrient standards under consideration for rivers, streams, lakes, and reservoirs, and to meet the goal for a reduced size of the hypoxic zone in the Gulf of Mexico, nutrient loading from the upstream watersheds will need to be reduced. Although reducing nutrient loadings from all watersheds throughout the MARB would provide a comprehensive way to achieve these goals, it would not be the most efficient strategy because not all watersheds contribute equal quantities of nutrients to local streams and downstream coastal waters, including the Gulf of Mexico (e.g., Alexander *et al.*, 2008). An alternative strategy might be to target or rank watersheds based on the number of locations identified as impaired by nutrients. The number of locations that were classified as impaired within each of the 818 eight-digit Hydrologic Unit Code (HUC8) (Seaber *et al.*, 1987) watersheds in the MARB is shown in Figure 1. Although this strategy identifies locations that the individual states have reported to be impaired by nutrients, this strategy has problems in an overall ranking scheme because each state has different criteria for identifying what locations are impaired, and it is uncertain to what extent these locations influence downstream waters.

A third strategy is to identify and rank the areas contributing the largest proportion of the total load and concentrate management action in these areas (Ouyang *et al.*, 2005; UW-CALS, 2005). Targeting reduction efforts on a landscape scale is worthwhile because unit-area nutrient export (expressed as kg/km^2^/year) from watersheds vary by up to several orders of magnitude among streams across the United States (U.S.) (Alexander *et al.*, 2008) and within smaller regions, such as states (Corsi *et al.*, 1997). Approaches have been developed for targeting management and other total maximum daily loads activities within small watersheds (e.g., Tomer *et al.*, 2003), but only a few studies have attempted to target watersheds at broad geographic scales (such as targeting specific river basins or specific states in the MARB) (Goolsby *et al.*, 1999; Ouyang *et al.*, 2005; Alexander *et al.*, 2008; Diebel *et al.*, 2009). Diebel *et al.* (2009) developed a predictive model to estimate the sediment and phosphorus load reduction that should be achievable following the implementation of riparian buffers in watersheds throughout Wisconsin, and used this information to rank the watersheds based on their potential load reductions. Ouyang *et al.* (2005) developed a GIS (geographic information system)-based erosion model for the Great Lakes tributaries to assess and compare their relative loadings of sediments, status of conservation practices, and their potential for further reductions to sediment and contaminant loadings. Goolsby *et al.* (1999) used multiple-regression techniques relating nitrogen inputs from various sources and measured nitrogen yields from 42 large drainage basins to estimate and rank nitrogen yields from all of the large river basins throughout the MARB. Alexander *et al.* (2008) used the Spatially Referenced Regression On Watershed attributes (SPARROW) model to estimate yields of total phosphorus (TP) and total nitrogen (TN) from small watersheds (median size of ∼60 km^2^) throughout the MARB to the Gulf of Mexico, and used this information to compare the relative contributions from each state.

Watersheds can be ranked on the basis of their respective loads, yields (by compensating for watershed size), incremental yields (that part generated in individual stream-segment drainages), or delivered incremental yields (by compensating for the loss of nutrients with transport from each watershed to the downstream receiving body) (Alexander *et al.*, 2008). Which of these characteristics should be used in the ranking procedure depends on the reason for the ranking. If one is most interested in the downstream receiving body, such as the Gulf of Mexico, then a ranking based on delivered incremental yield is appropriate. If one is more interested in delivery to local streams, then ranking based on incremental yields is appropriate.

Various types of models have been used to estimate loads and yields in large watersheds and can support the development and evaluation of watershed ranking strategies. These models range from simple statistical models, such as that in Goolsby *et al.* (1999) where TN yields were estimated as a function of several watershed attributes, to complex mechanistic simulation models, such as the Soil and Water Assessment Tool (Gassman *et al.*, 2003; Neitsch *et al.*, 2005) that simulate the primary hydrological, chemical, and biochemical processes in the soil and during the transport downstream. The SPARROW model (Smith *et al.*, 1997) has a hybrid statistical/mechanistic structure, which includes aspects of these two approaches and takes advantage of their strengths. All watershed models have estimation errors inherent in their predictions; however, these errors are rarely reflected in the watershed rankings that are developed from the model predictions of loads and yields. A more statistically rigorous and robust ranking would be to directly incorporate measures of the uncertainties in model predictions. For example, although a watershed may have a predicted load that is higher than several other watersheds, the loads (and thus the rankings) may not be statistically different. Thus, measures of the uncertainty in predictions of incremental and delivered yields are essential to develop reliable and informative ranking strategies. Although prediction uncertainties can be determined for mechanistic simulation models using parameter estimation methods (e.g., Doherty, 2004), these methods are difficult to use with complex, highly parameterized models (Beven, 2002) and may not incorporate all of the uncertainties in the model’s predictions. These parameter estimation methods are especially challenging to use in large watersheds and river basins, such as the Mississippi and its major tributaries, where mechanistic models are rarely applied. By contrast, prediction uncertainties are comparatively easy to generate using statistical models, such as SPARROW, which have been applied in large river basins, including the Mississippi and Atchafalaya (Goolsby *et al.*, 1999; Alexander *et al.*, 2008).

In this paper, we use results from a published SPARROW model (Alexander *et al.*, 2008) to estimate and rank TP and TN incremental yields from the 818 HUC8 watersheds throughout the entire MARB that are delivered to the Gulf of Mexico (for this analysis, taken to be the reach containing the U.S. Geological Survey monitoring station near St. Francisville, Louisiana). We describe and demonstrate robust statistical bootstrapping procedures to place confidence limits on nutrient yield predictions from SPARROW, and to place confidence limits on the individual ranks of the HUC8 watersheds. This information is then used to estimate the probability that each HUC8 watershed is among a collection of watersheds that contributes the largest quantities of nutrients to the Gulf of Mexico. We illustrate the application of the procedure to 818 watersheds in the MARB by identifying 150 watersheds that have the largest delivered nutrient yields (i.e., “top 150”). The selection of the number 150 is arbitrary and the method presented here could be applied to any user-selected number of watersheds. Results from this ranking procedure can be used by state and federal managers to target a set of “high priority” inland watersheds, where additional stream monitoring and evaluations of nutrient-reduction strategies could be undertaken. We also demonstrate how the results can be used by specific states to prioritize the target watersheds based on nutrient deliveries to local streams. This method of ranking is not a fully optimal approach to concurrent management of coastal and inland waters. Watersheds that rank near the lowest end of the spectrum in terms of nutrient delivery to the Gulf of Mexico, with management, can be expected to improve the water quality of local receiving waters. These local improvements may be of similar or greater value compared to the incremental improvements in the coastal receiving waters.

## Methods

### SPARROW Model

SPARROW is a GIS-based watershed model that uses a hybrid statistical/mechanistic approach to estimate nutrient sources, transport, and transformation in terrestrial and aquatic ecosystems of watersheds under long-term steady state conditions (Smith *et al.*, 1997; Alexander *et al.*, 2008). The present application of SPARROW includes nonconservative transport, mass-balance constraints, and water flow-paths defined by topography, streams, and reservoirs, based on a detailed stream-reach network (1:500,000 scale) with reach catchments (median size ∼60 km^2^) delineated from 1-km digital elevation models (Nolan *et al.*, 2002); the MARB contains ∼25,000 reaches. Given a specification of nutrient sources, the model is used to estimate nutrient delivery to streams from subsurface and overland flow (“land-to-water” delivery) in relation to landscape properties, including climate, soils, topography, drainage density, and artificial drainage. A brief description of the SPARROW model is given in the Appendix, and a detailed description of the model is given in Schwarz *et al.* (2006).

The SPARROW model was used to simulate the mean annual flux of TN and TP from each reach of the ∼25,000 reaches in the MARB as a function of ten nutrient sources (eight for TP), six climatic and landscape factors that influence land-to-water delivery (five for TP), and two factors describing nutrient removal in streams and reservoirs (Alexander *et al.*, 2008). Nutrient sources included atmospheric deposition of nitrogen, urban sources, and nutrients in the runoff and subsurface flow from agricultural and other lands. The population within the drainage area of each reach was used as a surrogate for all urban point and nonpoint sources of nutrients. Agricultural sources were separated according to their association with either cultivated croplands or nonrecovered manure. Cropland includes nutrient inputs from biological N_2_ fixation (soybeans, alfalfa, and hay), commercial fertilizer use on seven major crops, and animal manure that is recovered from confined animals on nearby farms and applied to crops as fertilizer. Nonrecovered manure pertains to nutrients derived from unconfined animals in pastures or losses from feedlots.

Model parameters for the sources, land-to-water delivery factors, and instream-decay terms were statistically estimated using weighted nonlinear least squares (WNLLS) regression, based on a calibration to the long-term mean annual loads (1975-1995) (Schwarz *et al.*, 2006) of TN and TP (i.e., the steady state response variables in the model) at 425 monitoring stations in the conterminous U.S. that were part of the National Stream Quality Accounting Network (Hooper *et al.*, 2001). Most of the stations used in the calibration have relatively large drainage areas: 90% of stations had drainages >820 km^2^. Mean annual loads for each station were standardized to the 1992 base-year to give an estimate of the mean nutrient load that would have occurred in 1992 if the mean flow conditions from 1975 to 2000 had prevailed (nutrient source inputs to the model are those for 1992). Although the model cannot be used to evaluate year-to-year changes, it also is not biased by the short-term meteorological variability that may occur throughout the study area in a given year. The specifics about the source and transport process specification, calibration, accuracy, and precision of the final models are presented in Alexander *et al.* (2008).

The 818 HUC8 watersheds in the MARB (median size = 3,400 km^2^) are used as the units for evaluating uncertainties in the rankings of SPARROW predictions of the nutrient incremental yields that are delivered to the Gulf of Mexico. The incremental nutrient yield (and delivered yield) was computed for each HUC8 watershed by summing the loads (or delivered loads) from each reach within the HUC8 watershed, and then dividing by the total area of the HUC8 watershed. The HUC8-level aggregation of the ∼25,000 reach-level SPARROW predictions in the MARB provides the most reliable spatial scale for evaluating the model predictions, one that is generally consistent with the spatial scale of the monitoring stations used to estimate the model parameters.

The model predictions of the incremental yields and delivered incremental yields that are used in the HUC8 ranking procedures reflect the simulated nutrient yields in circa 2002. The simulated yields account for changes from 1992 to 2002 in agricultural sources (biological N_2_ fixation in crops, farm fertilizer use, and crop harvesting); however, changes from 1992 to 1997 are used for animal manure nutrients and from 1990 to 2000 are used for population as described in Alexander *et al.* (2008). The coefficients of the calibrated model are unchanged for 2002 simulations; steady state conditions are also assumed based on long-term average streamflow over the 1975 to 2000 period. The predicted yields for 2002 only reflect changes in nutrient sources and are independent of any actual changes in streamflow from 1992 to 2002. The changes in agricultural sources also account for changes in the marginal rates of crop production from 1992 to 2002 (i.e., harvested biomass relative to nutrient inputs) (note that the estimated 1992 base-year model implicitly reflects crop production related to climatic conditions and farm practices and technologies in that year). The predicted yields for 2002 do not include the effects of any changes in farm management practices unrelated to crop fertilizer use and production or animal populations (e.g., addition of buffer strips or improvements in feedlot operations).

### Confidence Limits in SPARROW Predictions

The actual delivered fluxes from SPARROW are assumed to depend on a multiplicative error term that represents other sources and processes not included in the SPARROW analysis. Because of this residual term, and because the determination of predicted flux depends on coefficients that are estimated via statistical methods, the delivered yields and the ranking of those yields across HUC8 watersheds are subject to uncertainty. Because of the nonlinear manner in which the estimated coefficients enter the model, it was necessary to use bootstrap methods (Schwarz *et al.*, 2006) to assess the uncertainty. Bootstrap analyses were also used to correct for potential bias caused by log retransformations in the yield predictions, an additional consequence of the nonlinear model specification. A brief summary of the bootstrap method is presented here; a full description of the bootstrap methodology is provided in the Appendix to this paper.

The bootstrapping method was implemented by performing 200 repeated calibrations of the SPARROW model using randomly selected integer weights (which sum to 425, the total number of monitored reaches in both the TN and TP models) applied to each of the squared residuals at monitored reaches, resulting in 200 realizations of the estimated coefficients, yields, delivered yields, and residuals. The distribution of the estimated delivered yields for each reach from the 200 iterations was used to estimate the standard errors in the yields from SPARROW. The estimated confidence interval for delivered yields required explicit consideration of the distribution of residuals in the model, rather than just the summary statistical properties of the residuals. The bootstrap method for incorporating the distribution of the model residuals is based on the empirical distribution of the combined bootstrap-iteration estimate of the modeled component of delivered flux and a randomly selected weighted error from the original 425 monitored values obtained in the original calibration of the model. The 90% confidence interval for the delivered incremental yield from each HUC8 was then estimated using a ratio formulation of the hybrid bootstrap confidence limit (see Shao and Tu, 1995 and Schwarz *et al.*, 2006).

### Statistical Significance of the HUC8 Rankings

**FIGURE 2:**
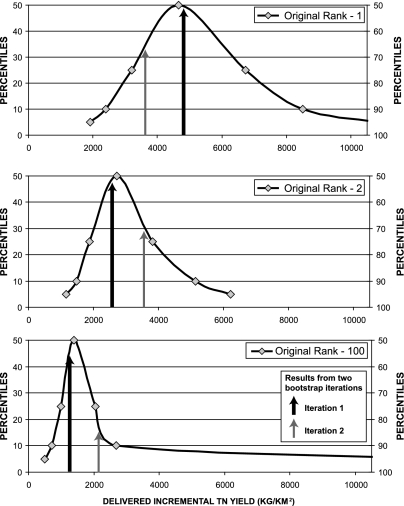
Distributions of Predicted Delivered Incremental Total Nitrogen (TN) Yields for Three HUC8 Watersheds From the Bootstrapping Procedure, With Two Selected Values From Specific Iterations.

The assessment of uncertainty in the ranks of the delivered yields was also based on bootstrap methods. Unlike the methods used to place confidence limits on specific SPARROW predictions, the theoretical basis behind the underlying assessment of uncertainty in ranks was less rigorous. The bootstrapping method was implemented by performing 200 repeated estimations of the delivered incremental yields from each HUC8 watershed. In each iteration, the delivered incremental yields were estimated by multiplying the modeled estimate of delivered incremental yield for each watershed by a randomly selected, exponentiated, weighted model residual from the 425 residuals estimated in the WNLLS regression calibration of the SPARROW model. With each bootstrap iteration, a different delivered incremental yield is obtained for each of the 818 HUC8 watersheds. These estimates are distributed around the delivered incremental yield obtained directly from the original SPARROW model and used in the original ranking (the distributions of these estimates are shown for three sites in Figure 2). After each bootstrap iteration, the delivered incremental yields from the 818 watersheds are ranked. Delivered incremental yields for two iterations of this process are shown in Figure 2. On the basis of the 200 different ranks for each HUC8 watershed (a different rank for each bootstrap iteration), it is possible to (1) determine with what certainty (probability) a specific watershed would be ranked in, or not in, the top specified number of watersheds and (2) estimate the confidence intervals of the rankings. A specific watershed would be placed in the top 150 contributing watersheds with 90% confidence, if 90% or more of the time (≥180 of the 200 bootstrap iterations), the watershed ranked ≤150, and would be placed out of the top 150 watersheds, if 90% of the time the watershed did not rank ≤150.

Confidence intervals were placed on the rankings for each HUC8 watershed on the basis of the percentiles of the distributions of the 200 different ranks from the bootstrapping procedure. The 5th and 95th percentile of the distributions of the ranking from the bootstrapping procedure represent the lower and upper bounds of the 90% confidence limits of the rankings for each watershed.

## Results

### Incremental Nutrient Yields by HUC8 Watershed

**FIGURE 3:**
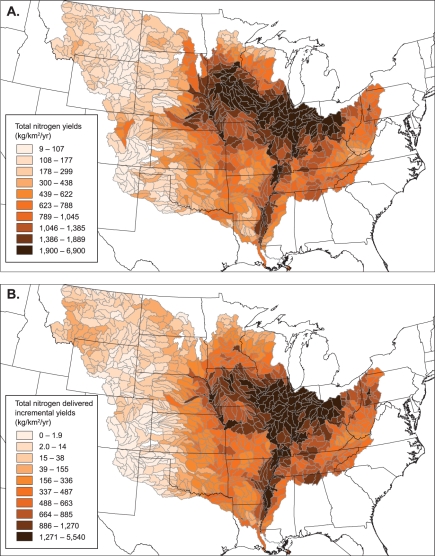
Distributions (deciles) of Incremental Yields (A) and Delivered Incremental Yields to the Gulf of Mexico (B) of Total Nitrogen (TN) for the HUC8 Watersheds Within the MARB for Conditions Similar to 2002.

**FIGURE 4:**
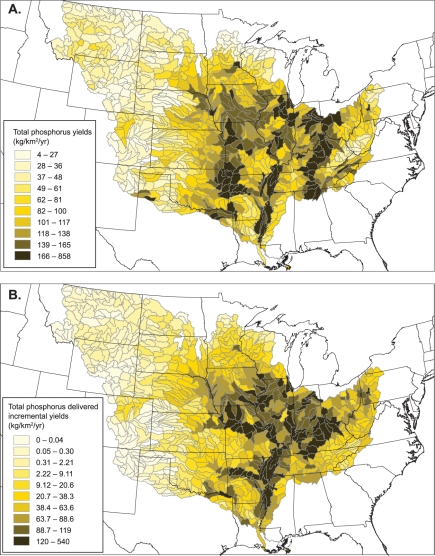
Distributions (deciles) of Incremental Yields (A) and Delivered Incremental Yields to the Gulf of Mexico (B) of Total Phosphorus (TP) for the HUC8 Watersheds Within the MARB for Conditions Similar to 2002.

Incremental TN and TP yields describe the mass of nutrients entering streams per unit area of the incremental drainages, and are mediated by the amount of nutrients supplied to the area and the climate and landscape properties that affect their delivery to nearby streams. Incremental yields from HUC8 watersheds in the MARB range from 9.4 to 6,900 kg/km^2^/year for TN (Figure 3A) and 4.0 to 858 kg/km^2^/year for TP (Figure 4A). Highest TN and TP yields are generally in similar regions and include many watersheds in the Central Mississippi, Ohio, and Lower Mississippi River basins. The highest TN yields closely coincide with intense agriculture in Indiana, Illinois, and Iowa, whereas highest TP yields are spread over a larger area (often associated with urban areas) and shifted southward. Differences in the geographic patterns in the yields can be primarily explained by differences in the types of agricultural and nonagricultural sources that contribute nutrients and the presence of major urban areas. Lowest yields for TN and TP are from watersheds in the western regions of the MARB, where streamflows are lower and inputs of nutrients are generally smaller.

Delivered incremental yield describes the amount of the incremental yield that is delivered to some downstream point, in this case the Gulf of Mexico, after accounting for the cumulative effect of aquatic removal processes (primarily denitrification for TN and deposition in reservoirs for TP). Delivered incremental TN yields from HUC8 watersheds range from 0 to 5,540 kg/km^2^/year (Figure 3B) and delivered TP yields range from 0 to 540 kg/km^2^/year (Figure 4B). Instream and in-reservoir losses result in much of the TN and TP delivered to the Gulf of Mexico originating from similar regions: primarily from watersheds in the Central Mississippi, Ohio, and Lower Mississippi River basins, especially those near large rivers. Lowest delivered incremental yields were from watersheds in the western regions of the MARB, where streamflow is lower, inputs are generally smaller, and longer river distances enhance instream removal.

Although the magnitude of the incremental yields and delivered incremental yields differ, the geographic patterns in incremental yields and delivered incremental yields for TN differ only slightly, as seen in the decile distributions in Figure 3; the highest delivered TN yields occur a little further east than the incremental yields. The largest differences in the patterns are the result of decreases in delivery from southern Minnesota and Iowa. There are larger changes in the patterns for TP than for TN. The largest changes in TP patterns are that the relatively higher delivered yields are closer to the main rivers, such as the Mississippi and Ohio Rivers, than were the yields themselves. The largest relative decreases in delivery occur in Arkansas, Tennessee, and Iowa, and are primarily caused by losses in reservoirs. The relative decreases can be best seen in eastern Tennessee (Figure 4), where the relatively large incremental TP yields are reduced by losses in large reservoirs.

### Ranking of HUC8 Watersheds by Nutrient Yields

**FIGURE 5:**
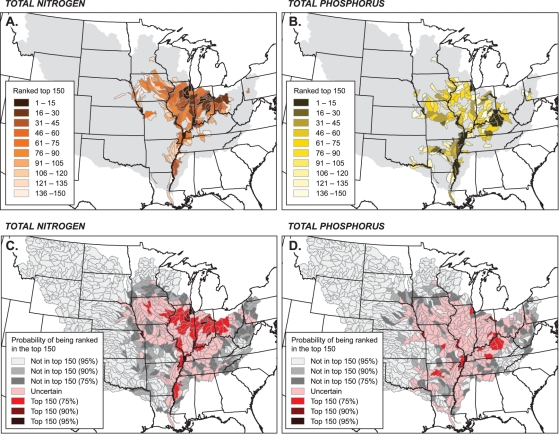
Map Showing the Top 150 HUC8 Contributing Watersheds Within the MARB to the Gulf of Mexico for TN (A) and TP (B) and Maps Showing the Certainty With Which the Watersheds are Placed In or Out of the Top 150 Contributing Watersheds for TN (C) and TP (D).

Ranking the HUC8 watersheds throughout the MARB by the predicted incremental yields or delivered incremental yields from SPARROW can be as straightforward as sorting the predicted yields and ranking them from 1 (highest yield) to 818 (lowest yield). All incremental yields and delivered incremental yields and their respective rankings are given in the Supporting Information material to this paper. The results of the ranking process are shown for delivered incremental yields for TN and TP in Figures 5A and 5B (only the top 150 watersheds are colored). Almost all of the top 150 watersheds are in the Corn Belt or near the Mississippi River, with the highest yields of TN being in northern Illinois and central Indiana and highest yields of TP being from watersheds along the Mississippi River, and in northern Kentucky, and distributed through Missouri, Illinois, and Indiana. The HUC8 watershed with the highest TN and TP incremental yields encompasses the area near Chicago. Therefore, if one were interested in placing management efforts only in a specified number of the highest contributing watersheds, the watersheds to place efforts could be readily identified.

**FIGURE 6:**
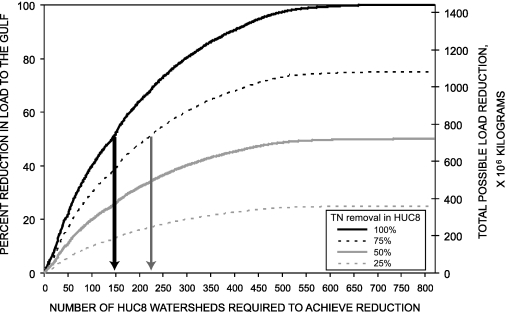
Number of HUC8 Watersheds Required to Reduce Various Percentages of the TN Load to the Gulf of Mexico as a Function of the Percent Removal From Each Watershed. Arrows indicate number of HUC8 watersheds to achieve a 50% reduction for 100 and 75% removal of TN.

After sorting the HUC8 watersheds on the basis of their delivered incremental yields and summing the yields from the highest ranked watersheds to the lowest, an accumulated yield plot can be created (Figure 6). From this figure, it is possible to determine the minimum number of watersheds required to reduce the total load to the Gulf of Mexico by a specified percentage or specified load in kg. For example, if one wanted to obtain a 50% reduction in TN loading to the Gulf of Mexico, it would require removing all the TN loading from the top ∼150 HUC8 watersheds. If it were assumed that only 75% of the delivered load could be removed from each watershed, then it would require that ∼225 watersheds be included to obtain a ∼50% reduction. If only 50% of the delivered load could be removed from each watershed, then it would require that all of the 818 watersheds be included.

### Effects of Uncertainty on the Ranking

**FIGURE 7:**
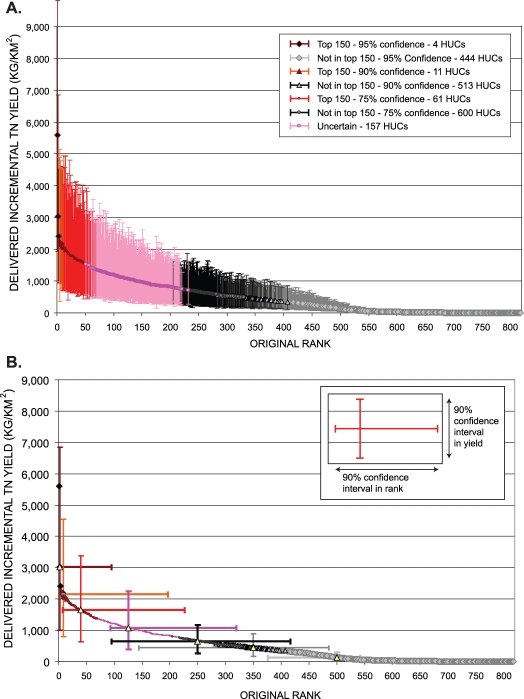
Delivered Incremental TN Yields as a Function of the Rank of the HUC8 Watershed (A), 90% Confidence Limits (vertical bars) Are Placed on Each Prediction. The value for each watershed is color coded based on the certainty it is in or not in the top 150 contributing watersheds. (B) Illustration of the 90% confidence limits (horizontal bars) on the ranks of six selected HUC8 watersheds; the 90% confidence limits on the predictions (vertical bars) are also given.

Differences in the delivered incremental yields from many of the HUC8 watersheds are quite small, especially if the first few watersheds with the largest difference in yields (Figure 7) are not included; therefore, small differences in predicted yields can have a large impact on the ranking of the HUC8 watersheds. To determine how confident we are in the predicted yields from each watershed, 90% confidence limits on each prediction from SPARROW were computed (Figure 7A). The 90% confidence limits for the prediction of the yields from any specific HUC8 watershed were quite large, typically within a factor of 2-3 of the original value. To determine how the uncertainty in the predicted yields affect the ranking of the HUC8 watersheds, a bootstrapping procedure was conducted to incorporate the uncertainty in the predicted yields into the ranking process (see the Methods Section). As a result of this process, 90% confidence limits were placed on the ranks themselves (the 90% confidence limits of the ranks are shown as horizontal bars for six example sites in Figure 7B). The 90% confidence limits on the ranks demonstrate that because of the uncertainties in the SPARROW algorithms, our certainty in the specific ranking of any HUC8 watershed is quite wide. For example, the 90% confidence in the ranking of the Upper Wabash River watershed in Indiana and Ohio, which was originally ranked eighth with respect to delivered incremental TN yields, ranges from 8 to 227, and the Pecatonica River watershed in Illinois and Wisconsin that originally ranked 125, ranges from 26 to 320. The original ranking for the delivered incremental yields for TP and TN and their respective 90% confidence limits are given for each HUC8 watershed in the Supporting Information material to this paper.

Having confidence limits in the yields and the rankings of the yields, rather than having specific values, makes it much more difficult to determine, which HUC8 watersheds to suggest to implement management efforts in, if only a selected number of contributors are to be considered. For example, if we want to identify which HUC8 watersheds are in the top 150 contributors to the Gulf of Mexico (the approximate number of watersheds for which loads would have to be totally removed to obtain a 50% reduction in the TN load), this uncertainty should be considered. Given the wide 90% confidence intervals in the yields, many of the watersheds that originally ranked near 150, may or may not actually be in the top 150. One approach to incorporate this uncertainty is to use the information from the bootstrapping procedure, which was used to place confidence intervals in the original ranking (Figure 7B). If a watershed ranked in the top 150 in ≥95% bootstrapping iterations, then we can say the watershed is in the top 150 with 95% confidence. If a watershed were in the top 150 in ≥90% of the bootstrap iterations, then it would be in the top 150 with 90% confidence, and so on. Similarly, if a watershed ranked in the top 150 in <5% of the bootstrap iterations, then it would not be in the top 150 with 95% confidence. If a watershed ranked in the top 150 in <10% of the bootstrap iterations, then it would not be in the top 150 with 90% confidence, and so on.

This information was then used to determine which watersheds could be considered to be in the top 150 for delivered incremental yields of TN and TP to the Gulf of Mexico if uncertainty is accounted for in the assessment (Figures 5C and 5D). With 95% confidence, we can identify four HUC8 watersheds for TN and one watershed for TP that are in the top 150 contributors. With 90% confidence, we can identify 11 watersheds for TN and three watersheds for TP that are in the top 150 contributors (Table 1). Most of the watersheds that we can confidently say (with ≥75% confidence) are in the top 150 are in Indiana, Illinois, and Kentucky. Most of these watersheds have little urban inputs, except the HUC8 watersheds containing Chicago and Memphis. We can, however, confidently identify many more watersheds that are not in the top 150. With 95% confidence, we can identify 444 watersheds for TN and 459 watersheds for TP that are not in the top 150 contributors, and with 90% confidence, we can identify 513 watersheds for TN and 505 watersheds for TP that are not in the top 150 contributors (Table 1). Most of the watersheds that are not in the top 150 contributors (with ≥75% confidence) are in the western part of the MARB, where there is relatively low runoff, or in Minnesota, Wisconsin, or in the eastern part of the MARB where mostly forested areas exist. There are more watersheds that we can say with confidence are in the top 150 for TN than for TP because of the more accurate TN SPARROW predictions and the wider range in TN yields than TP yields. The distributions of the HUC8 watersheds that we can say with 75% confidence are in and not in the top 150 for TN are similar to those for TP. The HUC8 watersheds that are classified as possibly being in the top 150 are those in areas that have been found to have the highest yields by Goolsby *et al.* (1999). How confidently we can classify whether each watershed is in the top 150 and the relative importance of urban areas are given in the Supporting Information material to this paper.

**TABLE 1:** Number of HUC8 Watersheds In or Not in the Top 150 Contributors to the Gulf of Mexico as a Function of the Confidence in the Classification.

Confidence Limits (%)	Total Nitrogen	Total Phosphorus
Number of HUC8 watersheds in the top 150		
95	4	1
90	11	3
75	61	22
Number of HUC8 watersheds not in the top 150		
95	444	459
90	513	505
75	600	573

Note: HUC8, Eight-digit Hydrologic Unit Code.

Even with the relatively large confidence limits presented here, the results from SPARROW can be very useful in the ranking process for HUC8 watersheds in the MARB or other smaller areas. For example, of the 818 watersheds in the MARB, only 218 for TN and 245 for TP are considered to possibly be in the top 150, the remaining 444 (for TN) and 459 (for TP) watersheds statistically rank above 150 and, therefore, can be removed from special consideration (with 95% confidence), or 600 (for TN) and 573 (for TP) watersheds can be removed from special consideration with 75% confidence. Therefore, this information can be used to remove many areas from special consideration with respect to their importance of their contributions to loading to the Gulf of Mexico.

## Discussion

### Ranking Over Different Geographical Scales

**FIGURE 8:**
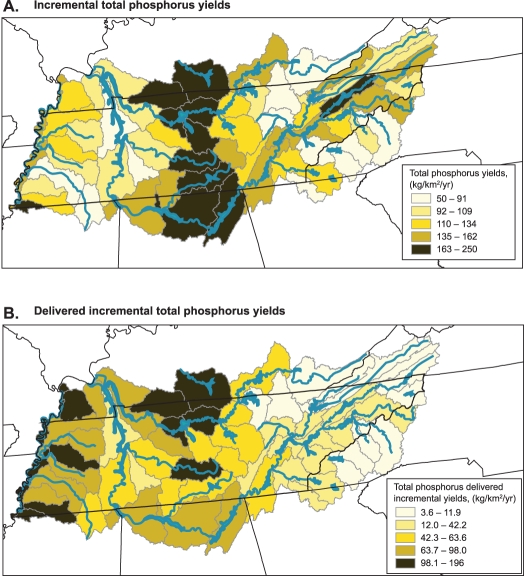
Distribution (deciles) of the Incremental Total Phosphorus (TP) Yields (A) and Delivered Incremental TP Yields (B) for the HUC8 Watersheds Within Tennessee.

The ranking discussed thus far has focused on the importance of export from specific HUC8 watersheds to the overall loading to the Gulf of Mexico. Actual implementation and prioritization of efforts, however, are often done at a smaller scale, such as in a specific state. An example of the ranking results for a given state, Tennessee, is shown in Figure 8. If water-quality managers in Tennessee are most interested in decreasing the load to some downstream location, such as the Gulf of Mexico, they could examine the delivered incremental yields as previously discussed. If water-quality managers are most interested in improving the water-quality in local streams and reservoirs, however, they may want to target or identify the watersheds based on the nutrients delivered to local streams. In this case, they would examine the incremental yields. Which of these conditions managers are most interested in improving, affects the prioritization process.

If water-quality managers are most interested in decreasing the number of locations that were identified as impaired (Figure 1), they would probably want to compute the incremental yields from each of their HUC8 watersheds and then rank them (Figure 8A). If they are primarily interested in reducing the TP load to the Gulf of Mexico, they would rank the delivered incremental yields (Figure 8B). These two yields can be quite different. In the case of TP, the differences in the yields and ranking for the eastern and central parts of Tennessee were primarily caused by the deposition of TP in large reservoirs. This information can assist managers in prioritizing efforts to improve local streams and downstream coastal waters, such as the Gulf of Mexico; however, the final overall ranking will need to consider the relative importance of improving local problems relative to downstream problems.

### Statistical Models *vs*. Mechanistic Models

Statistical models require an extensive dataset of stream monitoring sites with information for both the dependent (average annual loads) and independent variables (nutrient sources and environmental variables), whereas mechanistic models (i.e., without statistically estimated parameters) can be used to simulate water-quality conditions with only very limited stream monitoring data. A more extensive dataset, however, is required to characterize the complex set of processes and state conditions in applications of mechanistic models to large river basins. Hybrid statistical/mechanistic models, like SPARROW, share many of the benefits of mechanistic simulation models but also allow confidence limits to be reliably estimated for the model’s predictions and watershed rankings. The confidence limits on the individual predictions from SPARROW are generally large but incorporate several sources of error, including error in the estimations of the model due to the reliance on a finite sample, and structural error in the prediction algorithms due to factors that are not accounted for in the model. Most mechanistic models, on the other hand, simulate specific processes, but each simulation usually only has one prediction for a given location and confidence limits can be difficult to estimate. Therefore, the specific rankings provided by many mechanistic models may provide a false sense of accuracy in the final ranking among watersheds. Statistical approaches have been developed that attempt to quantify the confidence limits in the predictions from mechanistic models. For example, parameter estimation methods, such as in the Parameter Estimation model (PEST) (Doherty, 2004), can be applied to mechanistic models to estimate uncertainties associated with the model coefficients and predictions and hence provide an estimate of the confidence intervals for predictions of nutrient yield. However, it is difficult to reliably quantify the structural errors of mechanistic models without detailed calibration datasets (Moore and Doherty, 2005), which are especially difficult to obtain for large watersheds and river basins. Only with the confidence limits incorporated into the final predictions from watershed models can one determine if the small simulated differences in nutrient yields and rankings among watersheds are real and should be used to prioritize the targeting of watersheds for nutrient management.

### SPARROW Modeling at Smaller Regional Scales

The results presented in this paper were based on SPARROW models calibrated using 425 sites distributed throughout the U.S. (Alexander *et al.*, 2008). These models enabled yields and confidence limits in these yields to be estimated for rivers throughout the country. Within the MARB, there was ∼2.5-3 orders of magnitude variation in the incremental TN and TP yields, with even a wider range in the delivered incremental yields, whereas, the 90% confidence in the predictions were only within a factor of 2-3 of the predicted value. These predictions, with the specified confidence ranges, enable general management decisions to be made; however, the certainty in the predicted values and the ranking may not be adequate to guide local decisions. If regional SPARROW models were developed that incorporate additional water-quality data from other federal, state, and local agencies, especially from small watersheds, and incorporate better defined watershed attributes, such as point source data, then more specific sources and delivery terms may be determined. Finer scale regional SPARROW models may enable yields to be quantified more accurately at smaller than a HUC8 scale, and should result in smaller errors in model coefficients, narrower confidence limits on predictions, and enable a more precise watershed ranking process.

## Conclusions

To reduce the size of the hypoxic zone in the Gulf of Mexico and improve the water quality of rivers, streams, and other receiving water bodies, management efforts must be implemented at specific locations in the landscape to reduce nutrient runoff. The methods described herein could be applied to identify and target management efforts (including additional stream monitoring) in the areas producing the highest nutrient yields. Various types of numerical models can be used to predict yields over large geographical areas, and it is a straightforward process to use the output from these models to rank or prioritize management efforts. However, all numerical models have estimation errors in their predictions; these uncertainties should be considered in efforts to prioritize watersheds for management. Whereas, it is very difficult to estimate the overall uncertainty in many mechanistic models; it is comparatively easier to estimate the overall uncertainties in the predictions from statistical models.

In this paper, we used results from the hybrid statistical/mechanistic watershed model, SPARROW, to estimate delivered incremental yields from the 818 HUC8 watersheds in the MARB and then rank the watersheds based on their respective yields. Within the MARB, there was ∼2.5-3 orders of magnitude range in the incremental yields, with even a wider range in the delivered incremental yields, whereas, the 90% confidence in the predictions were only a factor of 2-3 of the predicted values. The highest TN and TP incremental yields and delivered incremental yields were generally in similar regions of the MARB: watersheds in the Central Mississippi, Ohio, and Lower Mississippi River basins. A robust statistical bootstrapping procedure was presented, which uses the uncertainties in the nutrient yield predictions (from SPARROW), to place confidence limits on the individual ranks. Information from the bootstrapping procedure was then used to estimate the probability that each HUC8 watershed is among a collection of watersheds that contributes the largest quantities of nutrients (e.g., “top 150”) to the Gulf of Mexico. Because of the wide confidence intervals in the predictions from SPARROW, only a few watersheds could be placed into the top 150 with statistical certainty; however, many more watersheds could be removed from consideration of being in the top 150. This ranking procedure was also demonstrated for use by a specific state to prioritize the targeting of watersheds for local improvements in streams and reservoirs.

The information presented here for the MARB could assist managers in prioritizing management efforts based on improving nutrient conditions in local streams and improving nutrient conditions in downstream coastal waters, such as the Gulf of Mexico. This method of ranking is not a fully optimal approach to concurrent management of coastal and inland waters. For example, watersheds that rank near the lowest end of the spectrum in terms of nutrient delivery to the Gulf of Mexico, with management, can be expected to improve the water quality of local receiving waters. These local improvements may be of similar or greater value compared to the incremental improvements in coastal receiving waters.

## Appendix

### Brief Description of the SPARROW Model

SPARROW is a GIS-based watershed model that uses a hybrid statistical/mechanistic approach to estimate nutrient sources, transport, and transformation in terrestrial and aquatic ecosystems of watersheds under long-term steady state conditions (Smith *et al.*, 1997; Alexander *et al.*, 2008). SPARROW includes nonconservative transport, mass-balance constraints, and water flow-paths defined by topography, streams, and reservoirs, based on a stream-reach network with delineated reach catchments. The model-estimated flux leaving each reach (*i)* in SPARROW, , is given by

(A1)

The first summation term represents the flux () from all upstream confluent reaches  that are delivered downstream to reach *i*. *δ*_*i*_ is the fraction of upstream flux delivered to reach *i*; *δ*_*i*_ generally equals 1 unless the upstream end of reach *i* is the location of a diversion.  is the stream transport function representing attenuation processes acting on flux as it travels along the reach pathway (instream decay). This function defines the fraction of the flux entering reach *i* at the upstream node that is delivered to the reach’s downstream node. The factor is a function of measured stream and reservoir characteristics, denoted by the vectors **Z**^*S*^ and **Z**^*R*^, with corresponding coefficient vectors **θ**_*S*_ and **θ**_*R*_.

The second summation term represents the incremental flux (that is introduced to the stream network in reach *i*). This term is composed of the flux originating from specific sources, indexed by *n* = 1,…, *N*_*S*_. Associated with each source is a source variable, denoted *S*_*n*_. Depending on the nature of the source, this variable could represent the mass of the source available for transport to streams, or it could be the area of a particular land use. The variable *α*_*n*_ is a source-specific coefficient that converts source variable units to flux units. The function  represents the land-to-water delivery factor. For sources associated with the landscape, this function along with the source-specific coefficient determines the amount of a constituent delivered to streams. The land-to-water delivery factor is a source-specific function of a vector of delivery variables, denoted by , and an associated vector of coefficients **θ**_*D*_. The last term in the equation, function , represents the fraction of flux originating in and delivered to downstream end of reach *i*. This term is similar in form to the stream delivery factor defined in the first summation term but is used to transport flux only from the midpoint of the reach to the outlet. A more in-depth description of the SPARROW model is given in Schwarz *et al.* (2006).

### Confidence Limits in SPARROW Predictions

The actual delivered flux from SPARROW is assumed to depend on a multiplicative error term added to Equation (*A*1), which represents other sources and processes not included in the SPARROW analysis. Because of this residual term, and because the determination of the predicted flux depends on coefficients that are estimated via statistical methods, the estimated delivered yield, and the ranking of that yield across HUC8s is subject to uncertainty. Due to the nonlinear manner in which the estimated coefficients enter the model, it was necessary to use bootstrap methods to assess the uncertainty in the yields from SPARROW and the ranking based on the yields.

Delivered yield for each HUC8 is estimated by summing the incremental flux component of Equation (*A*1) across all reaches within a HUC8 watershed, and then dividing the sum by the area of the HUC8 watershed. To simplify notation, let **β** represent the set of all model coefficients, , and let  denote the incremental flux for reach *i*, that is,  corresponds to the second summation term and factor  in Equation (*A*1).  is then defined as the fraction of flux leaving reach *i* that is delivered to the Gulf of Mexico and is formed by the product of the delivery factor for all reaches in the flow path between reach *i* and the mouth of the Gulf of Mexico. The *modeled* component of delivered yield for HUC8 watershed *u* is given by the aggregation of delivered incremental flux across all reaches in the unit, , normalized by the cataloging unit area, *A*_*u*_. *Actual* delivered yield, denoted *Y*_*u*_, is composed of modeled delivered yield and a multiplicative model error term,

(A2)

where *ε*_*u*_ is an independent, identically distributed error term, assumed to have the same statistical distribution as the weighted residual from the estimated SPARROW model. Note that this formulation of the model error, consistent with the prediction methodology used in SPARROW (see Schwarz *et al.*, 2006), assumes the error term does not accumulate across reaches, but is simply “tacked-on” to the modeled component to account for unexplained variation in flux. The use of a multiplicative error is consistent with the assumption, and general observation, that the error process in a SPARROW model is scale-independent (see Schwarz *et al.*, 2006, for additional discussion).

Equation (*A*2), the amount of delivered yield from a HUC8 watershed, depends on unknown values of the model coefficients, **β**, and the error term, *ɛ*_*u*_. The calibrated SPARROW model provides WNLLS estimates of the coefficients, . Additionally, the weighted residuals from the model, obtained by comparing monitored and predicted flux at the *N*_*M*_ monitored reaches [denoted by the set ] and normalized by the leverage for the observation, *h*_*i*_, can be used to form a mean estimate of an exponentiated error term,

(A3)

where  is the estimated residual for monitored reach *i*, and *w*_*i*_ is the weight applied to the observation (only the TN model received non-unitary weights) (see Alexander *et al.*, 2008 and Schwarz *et al.*, 2006, for details). The WNLLS estimate of delivered yield is given by

(A4)

The nonlinearity of the  and  functions with respect to the estimated coefficients, , causes the WNLLS estimate of predicted yield given in Equation (*A*4) to be biased. A bootstrap procedure, described in greater detail in Schwarz *et al.* (2006), is applied to correct for this bias. The method is implemented by repeated estimation of the SPARROW model using randomly selected integer weights (that sum to *N*_*M*_), resulting in *R* realizations of the estimated coefficients and estimated mean exponentiated residual, denoted  and , *r* = 1,…, *R* (note that the integer weights used to implement the bootstrap method are not included in the weights, *w*_*i*_, used to compute the mean exponentiated weighted residual via the bootstrap iteration-specific computation of Equation *A*3 for the TN model). Accordingly, the *r*^th^ bootstrap-iteration estimate of delivered yield is given by

(A5)

*R* equals 200 for all bootstrap estimates derived in the present analysis. The bias-corrected estimate of delivered yield, denoted , is obtained using a ratio formulation of the bootstrap and WNLLS estimates

(A6)

The standard error of the bias-corrected estimate of delivered yield is given by (see Schwarz *et al.*, 2006, for a derivation)

(A7)

where  is the standard variance operator,

The estimate of the confidence interval for delivered yield requires explicit consideration of the distribution of the model residuals, rather than just the statistical properties of the exponential residuals as was used to derive the bias-corrected and standard error estimates. The bootstrap method for incorporating the distribution of the model residuals is to determine the empirical distribution of the combined bootstrap-iteration estimate of the modeled component of delivered flux and a randomly selected, exponentiated weighted error from the original *N*_*M*_ values obtained with the WNLLS model estimation (*N*_*M*_ equals 425 for both the TN and TP applications described in this paper). Let  denote the *r*^th^ bootstrap-iteration random selection from among the *N*_*M*_ WNLLS estimates of , . As explained in Schwarz *et al.* (2006), the *R* values of

(A8)

are set in ascending order. Let *P*_*c*_ denote the confidence interval coverage probability (set to 0.9 for this analysis), let *q*_*lo*_ correspond to the  ranked value, and let *q*_*hi*_ represent the  ranked value ( and  represents the next lowest and next highest integer, respectively). The resulting 90% confidence interval, known as an equal-tailed hybrid confidence interval (Shao and Tu, 1995), is given by

(A9)

### Significance Level and Confidence Limits in Rankings of the HUC8 Basins

The assessment of uncertainty in the ranking of the delivered yields was also based on bootstrap methods. Unlike the methods used to place confidence limits on specific SPARROW predictions, the theoretical basis behind the underlying assessment of uncertainty in ranks was less rigorous. The *ad hoc* method used here is based on a bootstrap percentile approach to estimating a significance level and confidence interval (Shao and Tu, 1995). The bootstrap method is based on *R* repetitions of the generated quantity

(A10)

For each repetition, *r*, the rank of each estimated delivered incremental yield () is recorded. The empirical distribution of these 200 individual ranks is assumed to be similar to the true distribution of ranks given the information contained in the SPARROW model. The model residual component of , which represents the bulk of the uncertainty in the analysis, conforms well with this assumption. Then on the basis of the 200 different ranks for each HUC8 watershed from the bootstrapping, it is possible to determine with what certainty (probability) a specific HUC8 would be ranked in or not in the top specified number of watersheds. For example, a specific watershed would be in the top 150 with 90% confidence, if 90% or more of the time (≥180 times of the 200 repetitions), the watershed ranked ≤150.

Additionally, the 90% confidence limits of the rankings for each watershed can be estimated by the 5th and 95th percentiles of the 200 rankings from the bootstrapping procedure.

## SUPPORTING INFORMATION

Table S1. Ranking of HUC8 Watersheds in the Mississippi. Atchafalaya River Basin on the Basis of Total Nitrogen Yields, With Confidence Intervals.

Table S2. Ranking of HUC8 Watersheds in the Mississippi/Atchafalaya River Basin on the Basis of Total Phosphorus Yields, With Confidence Intervals.

Please note: Neither AWRA nor Wiley-Blackwell is responsible for the content or functionality of any supporting materials supplied by the authors. Any queries (other than missing material) should be directed to the corresponding author for the article.
